# Comorbidity “depression” in heart failure — Potential target of patient education and self-management

**DOI:** 10.1186/s12872-017-0487-4

**Published:** 2017-02-15

**Authors:** Renato De Vecchis, Athanassios Manginas, Ewa Noutsias, Carsten Tschöpe, Michel Noutsias

**Affiliations:** 1 Cardiology Unit, Presidio Sanitario Intermedio “Elena d’Aosta”, Naples, Italy; 2 Interventional Cardiology and Cardiology Department, Mediterraneo Hospital, Ilias Street 8-12, 16675 Glyfada, Greece; 3 Department of Psychiatry, Theodor-Wenzel-Werk, Berlin, Germany; 4 Department of Cardiology, Charité - Universitätsmedizin Berlin, Campus Virchow Klinikum (CVK), Berlin, Germany; 5 Deutsches Zentrum für Herz Kreislaufforschung (DZHK) — Standort Berlin, Charité, Universitätsmedizin Berlin, Campus Virchow Klinikum (CVK), Berlin, Germany; 6 Berlin Center for Regenerative Therapies (BCRT), Campus Virchow Klinikum (CVK), Berlin, Germany; 7 Department of Internal Medicine I, Division of Cardiology, Pneumology, Angiology and Intensive Medical Care, University Hospital Jena, Friedrich-Schiller-University Jena, Erlanger Allee 101, D-07747 Jena, Germany

**Keywords:** Comorbidities, Depression, Heart failure, Patient education

## Abstract

The progress of the pharmacological and device treatment of heart failure (HF) has led to a substantial improvement of mortality and rehospitalization. Further potential for improvement may be heralded in the post-discharge management of HF patients, including patient education for self-management of HF. The study by Musekamp et al. is among the first publications providing evidence that improvements in self-management skills may improve outcomes of HF patients. It is concluded that multimodal approaches addressing comorbidities in HF patients with novel concepts, and by optimal and specific HF management, including patient education, may ultimately contribute to substantial improvement of HF prognosis.

The article by Musekamp et al. [1] focuses on a humanitarian and medical problem which is truly impressive, namely, the self-management of heart failure (HF) patients. It is an issue that shares some similitudes with the self-management of diabetes mellitus. Albeit we are lacking reliable laboratory parameters of the homeostatic balance control, as opposed to the situation in diabetes, which assist the patient and the physician in the decision-making (i.e. the blood glucose and glycosylated hemoglobin), the approach of patient education in HF gained importance also in the current ESC guidelines for heart failure (HF), and has been implemented in the discharge management after acute heart failure (AHF) [2, 3]. However, we are lacking data on the efficacy of patient education on endpoints of HF (i.e. rehospitalization, mortality). This issue may be of importance, since several telemedicine approaches have not consistently confirmed improvement of prognosis for HF [4, 5]. Hence, the manuscript by Musekamp et al., is among the first studies providing important evidence on this issue [1].

In HF, the guide for adjusting drug doses and for deciding changes in therapeutic program includes a varied constellation of clinical signs and symptoms (dyspnea, orthopnea, pulmonary rales, nycturia, peripheral edema, jugular venous distention, hepato-jugular reflux). They corroborate and complement the valuable information arising from repeated measurements of body weight, urine output and serum natriuretic peptide, along with echocardiographic assessment, in particular the changes over time in left ventricular ejection fraction (LVEF) and/or expiratory inferior vena cava diameter, as well as the alterations in left ventricular global longitudinal strain using speckle tracking technique. In CHF home management, the recommended approach is not simply based on diagnosis and therapy, but it is also an educational approach involving the patient, as well as the patient’s family [6]. It is important to remember that one of the main reasons of fulfillment and satisfaction for a patient is the achievement of a condition of fair autonomy regarding his usual daily activities and engagements in social life.

The relationship between the perception of a satisfactory degree of autonomy and the improvement of depressive symptoms in the CHF patient is underlined by Musekamp et al. [1]. Considerable frustration and discouragement may arise from the inability to perform normal activities of daily living, such as washing, dressing, going upstairs or driving the car. However, before soliciting training programs trying to retrieve these skills, it would be important to weigh the pros and cons, in particular to consider the risks related to the execution of relatively complex tasks that may put at further risk the patient’s health, or cause embittering disillusionment [7, 8]. There is growing transnational interest in education and self-management programs for patients with chronic illnesses [9, 10]. Today, a large repertoire of organizations including hospitals and community-based facilities are actively engaged in programs providing theoretical and practical bases for HF patient’s education and rehabilitation. In particular, the programs include techniques to decrease symptoms, to successfully antagonize distress, and to improve coping. The ultimate scheduled outcome for individuals attending these programs is improvement of the quality of life. Patient empowerment and self-management have also been found to be key intermediate outcomes.

Among the studies that deal with the topic of the empowerment of CHF patients, the one by Musekamp et al. [1] provides numerous relevant insights concerning rehabilitation practice. In particular, as regards self-reported self-management skills measured at the end of rehabilitation stage (duration 3 weeks), this variable predicted long-term improvement (after a 12-month follow-up) in physical quality of life. Moreover, the same variable predicted intermediate – term improvement in mental quality of life. Surprisingly, self-reported self-management skills after 3 weeks of rehabilitation were not able to predict neither intermediate nor long term changes in depressive symptoms. Conversely, self-reported self-management skills measured after a six-month follow-up were associated with favorable changes in quality of life and depressive symptoms estimated after 12 months.

Based on these findings, one would infer that, following a program of patient empowerment and functional rehabilitation specifically projected for CHF patients, an improvement in physical and mental quality of life may be achieved more rapidly compared to the target of earning an effective relief from depressive symptoms. Therefore, the educational and psychological support of dedicated professionals (specialized nurses, psychologists, sociologists) should also be continued beyond the term of the ordinary residential rehabilitation period.

All efforts should be made to provide the patient with an adequate educational support. For example, it would be very valuable the choice to enable the patient to understand the importance of practicing regular international normalized ratio (INR) measurements for patients under phenprocoumon oral anticoagulation. Additionally, the knowledge to independently perform adjustment of diuretics may be advantageous [11]. Similarly, it would be a wise initiative to inform the patient about the sometimes deleterious but well treatable comorbidities (from anemia to osteoporosis until prostatic hypertrophy), or the decision to enable him to recognize an incipient state of hemodynamic congestion based on weight gain or decrement of urine excretion. Likewise, it would be an appropriate educational strategy convincing the patient to comply with the precaution to practice prophylaxis with influenza vaccination annually, and to inform him about the risks related to prolonged immobility in case of bone fractures or sural thrombophlebitis [2].

The increased awareness of possible disturbances and workable remedies can elicit a sense of greater peace of mind, which in turn could aid the patient to better accept his condition of disability without becoming a victim of the anxiety and depression. The neutral results of escitalopram treatment of depressive CHF patients in the randomized controlled MOOD-HF trial [7] gave rise to the hypothesis that comorbidities such as depression, which undoubtedly contribute to outcome in HF, are not necessarily responsive to the standard treatment as the respective comorbidities per se. This notion is supported by the significant “antidepressive side effect” of left ventricular assist device (LVAD) treatment in advanced HF patients, which does not primarily target depression, but directly depressed systolic LV dysfunction [8]. A likewise discrepancy may be also attributable to the negative results of the SERVE-HF trial: the fact that adaptive servo-ventilation is helpful for central sleep apnea per se, does not necessarily mean that it is of advantage in central sleep apnea in HF patients [12]. A multimodal approach addressing comorbidities in HF patients with novel concepts, and by optimal and specific HF management, including patient education, may ultimately contribute to substantial improvement of HF prognosis [2]. We still have to pave a long way of standardization of patient education and self-management for this goal in HF management, and should encourage new studies for this rather neglected field of HF research.

## Abbreviations

AHF: Acute heart failure
CHF: Chronic heart failure
HF: Heart failure
INR: International normalized ratio
LVAD: Left ventricular assist devices
